# Areal Surface Roughness of AZ31B Magnesium Alloy Processed by Dry Face Turning: An Experimental Framework Combined with Regression Analysis

**DOI:** 10.3390/ma13102303

**Published:** 2020-05-16

**Authors:** Honghong Gao, Baoji Ma, Ravi Pratap Singh, Heng Yang

**Affiliations:** School of Mechatronic Engineering, Xi’an Technological University, Xi’an 710021, China; staticravi78@gmail.com (R.P.S.); yh752606754@163.com (H.Y.)

**Keywords:** surface roughness, magnesium alloys, machining, ANOVA, regression analysis

## Abstract

Surface roughness is used to quantitatively evaluate the surface topography of the workpiece subjected to mechanical processing. The optimal machining parameters are critical to getting designed surface roughness. The effects of cutting speed, feed rate, and depth of cut on the areal surface roughness of AZ31B Mg alloys were investigated via experiments combined with regression analysis. An orthogonal design was adopted to process the dry turning experiment of the front end face of the AZ31B bar. The areal surface roughness Sa and Sz of the end face were measured with an interferometer and analyzed through direct analysis and variance analysis (ANOVA). Then, an empirical model was established to predict the value of Sa through multiple regression analysis. Finally, a verification experiment was carried out to confirm the optimal combination of parameters for the minimum Sa and Sz, as well as the availability of the regression model for predicting Sa. The results show that both Sa and Sz of the machined end face reduce with the decrease in feed rate. The minimum of Sa and Sz reaches to 0.577 and 5.480 µm, respectively, with the cutting speed of 85 m/min, the feed rate of 0.05 mm/rev, and a depth of cut of 0.3 mm. The feed rate, depth of cut, and cutting speed contribute the greatest, the second and the smallest to Sa, respectively. The linear regression model can predict Sa of AZ31B machined with dry face turning, since the cutting speed, feed rate and depth of cut can explain 97.5% of the variation of Sa.

## 1. Introduction

Magnesium (Mg) alloys have been widely used in many industrial fields, such as electronics, automobile, and aerospace, due to their low density, high specific strength, and thermal conductivity, superior damping, and electromagnetic shielding performance [1,2]. Mg alloys are also extensively studied in the biomedical fields as potential implant materials, due to their excellent biocompatibility, biodegradability, and similar mechanical properties with bone [3,4,5]. When Mg alloys need to be machined as potential biomaterials, it is better to keep the surface from reaction with other substances during machining, since magnesium tends to have chemical reactions and forms hydrogen in contact with water-based coolants, which is extremely flammable. Additionally, oil-based lubricants introduce the danger of oil mist explosions [6,7]. Thus, the machining conditions should be carefully designed to keep the machining process safe and avoid surface deterioration. Moreover, the conventional machining process generally produces a low quality of surface integrity characterized by high surface roughness values [8]. Therefore, further surface modification is proposed to provide better surface integrity to satisfy the demand for the implant material. However, the machined surface topography influences the surface integrity induced by subsequent surface modification [9]. The machined surface topography affects the mechanical performance of contact interfaces in subsequent surface burnishing or rolling, as well as the biomechanical function in final use [10,11]. The dry machining surface topography of magnesium-calcium implant influences the surface integrity formed by surface burnishing [12]. Therefore, the good surface topography of Mg alloys is expected via surface machining, such as turning or milling. Surface roughness is always used to evaluate the surface topography after machining. Usually, the smaller the surface roughness, the better the surface topography. The influence of the machining parameters on the surface roughness needs to be carefully studied to optimize the machining process and get the expected surface topography. 

The machining process for the expected surface topography is complex because it is influenced by many factors, such as cutting conditions, cutting tool shapes and materials, cutting parameters, etc. In some researches, lubrication or coolants were used to take away the cutting heat during the machining process [13,14]. However, the surface of Mg alloys may react with the lubrication or coolants during machining, which will deteriorate the surface integrity and influence the subsequent study [15]. Thus, Mg alloys prefer to be machined in dry conditions to avoid surface deterioration in some conventional machining, such as turning and milling. 

Some researches focused on the effects of dry cutting parameters on surface roughness Ra. As one of the two-dimension (2D) parameters of surface roughness, Ra represents the arithmetic mean of the absolute values of the deviation of the surface profile from the mean line in the vertical direction [16]. A higher cutting speed combined with lower feed rate results in smaller surface roughness of MgCa3.0 subjected to the cylindrical turning process [17]. The surface roughness of AZ61 Mg alloy machined via cylindrical turning is influenced by the feed rate, followed by the cutting speed and depth of cut. The increase of the feed rate or the depth of cut leads to the increase of surface roughness, whereas the increase of the cutting speed leads to the decrease of surface roughness [18]. It is indicated that the machining parameters, such as cutting speed, feed rate, and depth of cut have synergetic effects on the surface roughness of Mg alloys [19]. 

Some researches were carried out to investigate the effect of main cutting parameters on surface roughness aiming to estimate optimal parameters and get designed surface roughness. To get surface roughness of UNSM11311 Mg alloy between 0.8 and 1.6 μm, the optimum cutting parameter for the feed rate is 0.15 mm/rev in dry turning [20]. For the intermittent turning of UNSM11917 Mg alloy, the feed rate was identified as the most significant factor and the cutting speed was not found significant on surface roughness (Ra and Rt) [21]. In another study based on Taguchi technology and statistical experiments, feed rate had the greatest effect on the surface finish of UNSM11311 by cylindrical turning [22]. The feed rate is the most important parameter to explain the surface roughness, while no clear influence was found for the cutting speed [23]. These researches show some significant effects of parameters on Ra in the cylindrical turning of Mg alloys. 

Although Ra remains the parameter of choice in a lot of papers about surface roughness, it is not the best representative of the surface quality because of the high dependence on the position of the measurements [24]. Therefore, there has been a drive towards three-dimension (3D) topographical characterization of surfaces with new standards defining area parameters [25]. The surface topography, alongside the 3D functional parameters, influence the performance of the machined surface [26]. Surface topography is 3D in nature, which can represent the natural characteristics of a surface and describe the surface more comprehensively. 

As one of the 3D characterization parameters of surface roughness, areal surface roughness Sa is most commonly used to quantitatively describe surface topography. It is the arithmetic mean of the absolute values of the surface deviations from the mean plane as described in (1), which is used to detect variations in the surface [27,28].

(1) $$\mathrm{Sa}=\frac{1}{A}\int_{0}^{x}\int_{0}^{y}\left|Z\left(x,y\right)\right|dxdy,$$

where *A* is the sampling area.

Sa is the average surface roughness evaluated over the complete 3D surface. It can provide visual images and the mechanism of surface formation of the surveyed surface [29]. For surface quality analysis, some other areal surface parameters were also used to further understand the feature of the surface. Sz is the peak to valley height of the areal surface as described in (2), which has an increased probability of finding the worst surface roughness. By comparing the line profile roughness parameters Ra and Rz with the areal equivalents Sa and Sz, Soady K.A., etc. found Sz is generally larger than Rz after processing tempered martensitic steel [30]. Therefore, Sz can be an indication of the worst case of a surface and a reference for setting up appropriate cutting parameters. It is indicated the greater probability of finding significant surface features using areal measurement than line measurement.

(2) Sz=Sp+Sv,

where Sp and Sv is the peak and valley value of the areal surface roughness, respectively.

Therefore, it is necessary to investigate the effects of cutting parameters on the areal surface roughness of the biomedical Mg alloy in dry face turning. Mg alloys with good surface quality could be prepared with proper machining parameters if effective experiment design and some optimization strategies were developed. Thus, in this research, the effects of dry turning parameters (cutting speed, feed rate, and depth of cut) on areal surface roughness (Sa and Sz) were investigated to evaluate the topography of the machined end face of AZ31B Mg alloy. First, the cermet cutting tool was selected and the face turning program was scheduled. The orthogonal experiment was designed and performed. The surface roughness of Sa and Sz was measured with an interferometer. Then, the influences of dry turning parameters on Sa and Sz were investigated by statistical analysis. The optimal combination of dry turning parameters was obtained based on the minimal value of Sa and Sz. A linear regression model was established to predict Sa of AZ31B machined with dry face turning. Finally, a verification experiment was carried out to confirm the optimal combination of parameters and the availability of the regression model for predicting areal surface roughness Sa. 

## 2. Materials and Methods

### 2.1. Workpiece Materials and Cutting Tools

Extruded AZ31B bars with a length of 100 mm and a diameter of 30 mm were used as the workpiece in this research. The front end face of the workpiece was machined with a dry turning method. The nominal chemical composition (wt.%) of the AZ31B is 2.96 Al, 0.52 Zn, 0.31 Mn, 0.16 Si, 0.006 Cu, 0.003 Fe, 0.001 Ni, and balance Mg. The presence of Al and Zn can improve the creep resistance of AZ31B. Although the biocompatibility of Al is limited, it seems to be a valid alloying element for AZ31B in body contact, since it can reduce the corrosion rate of the Mg alloys by stabilizing hydroxides in chloride environments [31]. Mg alloys with 9 wt.% of aluminum showed enhanced osteoblastic activity in the surrounding guinea pig femora. Small amounts of aluminum released continuously during the degradation process might be tolerable [32]. Therefore, the biosecurity of the presence of Al less than 3 wt.% in AZ31B is acceptable. 

Smaller surface roughness reaches when the cermet tool, compared with a cemented carbide tool, was utilized to machine stainless steel, due to the higher hardness, toughness, wear-resistance, and better thermo-stability [33]. Therefore, the ultrafine cermet tool (NX2525, Mitsubishi Corporation, Tokyo, Japan) was selected in this research for the continuous dry turning of AZ31B. The NX2525 is a triangular ultrafine cermet insert with a negative rake, 0° relief angle, and nose radius of 0.4 mm. Its hardness is up to 92.2 HRA and bending strength is up to 2.0 GPa [34]. Thus, it can be used in both low-speed and high-speed dry turning.

### 2.2. Machining Process and Experiment Design

**Figure 1:**
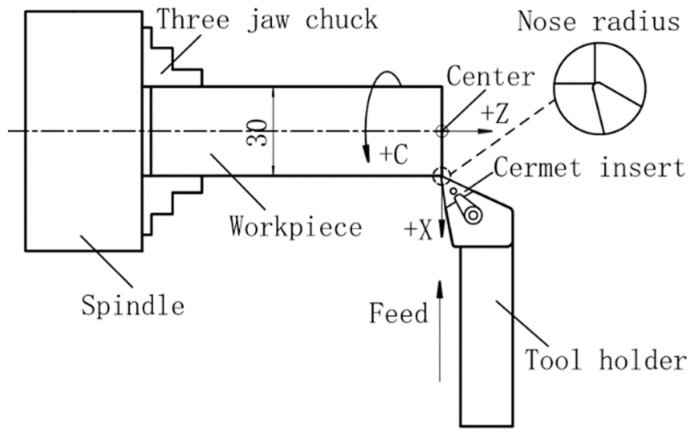
Schematic diagram of face turning.

The dry turning was performed on a three-axis lathe (CKD6136i, Dalian Machine Tool Group Co., LTD, Dalian, China) equipped with numerical control (NC) system. The workpiece is clamped by the three-jaw chuck. The maximum allowable rotational speed of the spindle is 3000 rpm. The cutting tool fixed on the rest can move along X and Z directions. In the face turning, the workpiece rotates with the spindle at a speed of no more than 3000 rpm. The cutting tool first moves the depth of cut from the end face to –Z direction and then feeds along –X direction until it reaches the center of the front end face of the workpiece. The schematic diagram of the face turning is shown in Figure 1.

In this experiment, cutting speed (factor A), feed rate (factor B), and depth of cut (factor C) were selected as the influence factors. Three levels were set for each factor, as shown in Table 1. In this face turning experiment, the cutting speed for each workpiece was kept constant at the set level by programming with NC code ‘G96’. The rotational speed rises with the feed movement and it was kept within 3000 rpm by using NC code ‘G50’. For example, ‘G96 S85’ was programmed to keep the linear cutting speed constant at 85 m/min. ‘G50 S3000’ was placed in front of ‘G96 S85’ to limit the rotational speed within 3000 rpm when the distance between the cutting point and the center of the front end face is smaller than 4.5 mm. Otherwise, the cutting speed will exceed 3000 rpm. Thus, there is a speed limit area on each end face, in which the cutting speed is smaller than the set level.

An orthogonal design method was utilized to organize the dry turning experiment. Thus, nine combinations of the parameters in the dry turning experiment are presented in Table 2. 

### 2.3. Areal Surface Roughness Measurement

**Figure 2:**
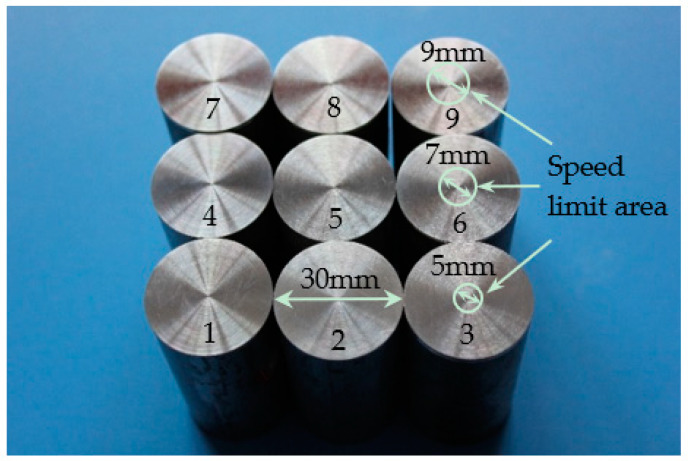
Image of the front end faces of samples 1 to 9.

The areal surface roughness parameters (Sa and Sz) are adopted to describe the surface topography of the AZ31B after dry face turning in this work. A white-light interferometer (Zegage Plus, Zygo, Middlefield, CT, USA) was used to measure the 3D surface roughness of the frontal surfaces of the samples after face turning. Zegage Plus is a 3D optical profiler with non-contact 3D coherence scanning interferometry (CSI). The optical resolution (transverse) is 0.95 µm with a 10X standard objective. The sampling area is 0.834 × 0.834 mm without additional filtering. Figure 2 presents the front end faces of samples 1 to 9. 

The white circles on samples 3, 6, and 9 indicate the speed limit areas corresponding to the cutting speed at 47, 66, and 85 m/min, respectively. Therefore, three sampling areas are selected outside the speed limit area of the frontend face of each sample, to make sure the cutting speed is consistent with the set value. The images of 3D surface topography and the values of Sa and Sz were obtained with the interferometer. By calculating the measured values of Sa and Sz from three sampling areas, the average of each sample was obtained, followed by the standard deviation (SD). The results are presented in Table 2.

The averages of Sa, as well as Sz, of samples 1 to 9 are different from each other, which attributes to the different combinations of the machining parameters. The smaller standard deviation means better consistency of the surface roughness of the three sampling areas. Thus, the consistency of Sa is better than that of Sz for each sample, due to the smaller standard deviation.

### 2.4. Multiple Linear Regression

Multiple linear regression is used to determine the linear relationship between one dependent variable and two or more independent variables. In this work, this method is utilized to find out the correlation coefficients between the areal surface roughness and the three machining factors, cutting speed, feed rate, and depth of cut, to establish a quantitative prediction model. The coefficient of the regression model is used as the goodness of fit to describe the degree of the predicted value fitting to the measured value. The closer the coefficient is to 1, the better the model fits.

## 3. Results and Discussion

### 3.1. Direct Analysis and Range Analysis

The smaller Sa and Sz indicate better surface topography. According to the averages of Sa and Sz of samples 1 to 9 in Table 2, the average of Sz nearly 10 times that of Sa indicates Sz can reflect some worst cases of the machined surface topography. The worst cases on the machined surfaces may be induced by the scratches of the breaking chips. Sample 3 (the combination of A_1_B_3_C_3_) has the worst surface topography, due to the maximal Sa and Sz (1.491 and 15.025 µm, respectively). Sample 4 (the combination of A_2_B_1_C_2_) has the minimal Sa (0.594 µm), while Sample 7 (the combination of A_3_B_1_C_3_) has the minimal Sz (6.923 µm). In this case, it cannot determine which one (Sample 4 or Sample 7) has a better surface topography, according to the averages of Sa and Sz. From a statistical point of view, the orthogonal experiment results need to be further analyzed to find a better combination of the parameters that maybe results in smaller Sa and Sz. Therefore, a direct analysis was used to seek for the optimal combination of the three factors. The results are presented in Table 3.

where $\bar{Ki}$ (i = 1, 2, 3) is defined as the arithmetic mean of Sa (or Sz) for each factor at each level i. The minimum $\bar{Ki}$ means the smallest surface roughness when a factor takes level i. Range value for a factor is the difference between the maximum and the minimum among $\bar{K1}$, $\bar{K2}$, $\bar{K3}$. Take Sa for example, $\bar{K1}$ for factor B (0.625 µm) is the arithmetic mean of 0.656, 0.594 and 0.624 µm ($\;\bar{K1_{B}\;}=\left(0.656+0.594+0.624\right)/3=0.625$). The range for factor B (0.842 µm) is the difference between the maximum ($\bar{K3_{B}\;}$) and the minimum ($\bar{K1_{B}\;}$). $\bar{K3_{A}}$,$\;\bar{K1_{B}}$, $\bar{K2_{C}}$ is the minimum among the $\bar{Ki}$ (i = 1, 2, 3) for factors A, B, and C, respectively. 

**Figure 3:**
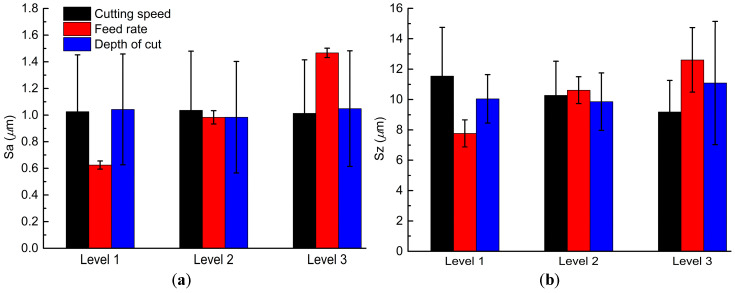
The influence of each level on the arithmetic mean of Sa (**a**) and Sz (**b**) for each factor.

The influence of each level on the arithmetic means ($i.e.,\bar{Ki}$) of Sa and Sz for each factor are depicted in Figure 3. The small variation of $\bar{Ki}$ for Sa shows with the increase of cutting speed and depth of cut from level 1 to 3 in Figure 3a. $\bar{Ki}$ of Sz decreases with the increase in cutting speed from level 1 to 3, as shown in Figure 3b. The increase of depth of cut leads to a slight decrease in $\bar{Ki}$ for Sa and Sz from level 1 to 2, and then an increase from level 2 to 3. The SD of the $\bar{Ki}$ of Sa, as well as that of Sz for the feed rate is smallest compared with that for the other two factors. It indicates the averages of Sa, as well as that of Sz, are well consistent with each other at the same feed rate.

It is worth noting that the $\bar{Ki}$ of Sa and Sz consistently increases with the increased feed rate from level 1 to level 3. Both the $\bar{Ki}$ of Sa and that of Sz reach to the smallest, when the cutting speed, feed rate, and depth of cut takes level 3 ($\bar{K3_{A}}$), level 1 ($\bar{K1_{B}}$), and level 2 ($\bar{K2_{C}}$), respectively. Therefore, the combination of A_3_B_1_C_2_ will probably result in smaller values of both Sa and Sz than that of A_2_B_1_C_2_ and A_3_B_1_C_3_. 

Range analysis is used to determine the importance of the three factors to the areal surface roughness Sa and Sz. In Table 3, the range of factor B is the largest (0.842 for Sa and 4.847 µm for Sz). It indicates that the feed rate has the greatest effect on both Sa and Sz among all the three factors. The range of factor A is the smallest (0.023 µm) for Sa, indicating cutting speed has the least effect on Sa. The range of factor C is the smallest (1.230 µm) for Sz, which means the depth of cut has the least effect on Sz. 

### 3.2. ANOVA Analysis 

Although range analysis can explain the importance of each factor on Sa and Sz, there is no objective criterion to evaluate the range. Thus, it is uncertain that the variation of the experiment results should be attributed to the variation of the factors or the random error. In this case, ANOVA analysis is used to investigate the significance of the three factors to the areal surface roughness Sa and Sz with the significance level of 0.05 (F_0.05_ (2, 2) = 19). If the F-value of a factor is larger than F_0.05_ (2, 2), or the *P*-value is smaller than 0.05, the factor has a significant effect on the result. The ANOVA analysis results of Sa and Sz are shown in Table 4.

The *P*-value of factor B is 0.0009 for Sa, which is much smaller than 0.05. It confirms that feed rate has a significant effect on the averages of Sa via dry turning process. The *P*-value of factor A for Sa is 0.5472 and that of factor C is 0.1099, which indicates that both cutting speed and depth of cut have a negligible effect on the averages of Sa. The *P*-values of factors A, B, and C are all much smaller than 0.05 for Sz, which indicates the three factors have significant effects on the Sz of AZ31B via dry turning process.

### 3.3. Regression Model of Sa

All the standard deviations and experimental errors of Sa are smaller than that of Sz in the above direct and ANOVA analysis. Thus, Sa is more appropriate for evaluating the whole surface topography and establishing a prediction model for surface roughness of AZ31B via dry face turning. The coefficients for evaluating the goodness of fit of the multiple regression model of Sa are listed in Table 5.

In Table 5, the coefficient *R* reaches to 0.992 with the standard error 0.058, indicating the model fits the data well. It can be used to predict the areal surface roughness of AZ31B by face turning in dry conditions. The adjusted *R^2^* is 0.975, which means the variation of the factors (i.e., cutting speed, feed rate, and depth of cut) can at least account for 97.5% variation of Sa. Then, ANOVA analysis was performed to test the validity of the regression model. 

The significance test is performed with the significance level of 0.05 and the F-value (F_0.05_ (3, 5) = 5.41). Table 6 shows the ANOVA analysis results of the regression model. The F-value of this model is 103.953, which is much larger than F_0.05_ (3, 5). It confirms that the regression model of Sa is valid. It also can be confirmed valid according to its significance (far less than 0.05). 

The coefficients of this multiple linear regression model are shown in Table 7. Therefore, the empirical model of Sa is described as (3).

(3) $$Y=-0.084-0.00035X_{A}+14.033X_{B}+0.028X_{C},$$

where dependent variable *Y* represents the areal surface roughness Sa (µm), independent variables *X*_A_, *X*_B_, and *X*_C_ represent the cutting speed (m/min), the feed rate (mm/rev), and the depth of cut (mm), respectively. The intercept (*B*_0_) is −0.084 µm. The non-standardized coefficient *B*_i_ (i = 1, 2, 3) means the predicted value of *Y* increases by *B*_i_ µm for every one unit increase of *X*_A_ (i = 1), *X*_B_ (i = 2), or *X*_C_ (i = 3). Therefore, the unit of the right side is the same as that of the left side in Equation (3).

The non-standardized coefficient cannot directly reflect the significance of each independent variable to the dependent variable, due to different units and ranges of the three independent variables. According to the standardized coefficients in Table 7, the feed rate can account for 99.2% of Sa. Cutting speed combining with the depth of cut only explains about 0.8% of Sa. The correlation of Sa with feed rate, depth of cut, cutting speed is the greatest, the second, the smallest, respectively. 

The *P*-value of feed rate is less than 0.05, which also indicates the feed rate is the most significant factor affecting Sa among the three factors. They are consistent with the previous conclusions drawn from range and ANOVA analysis. 

### 3.4. Verification Experiment 

**Figure 4:**
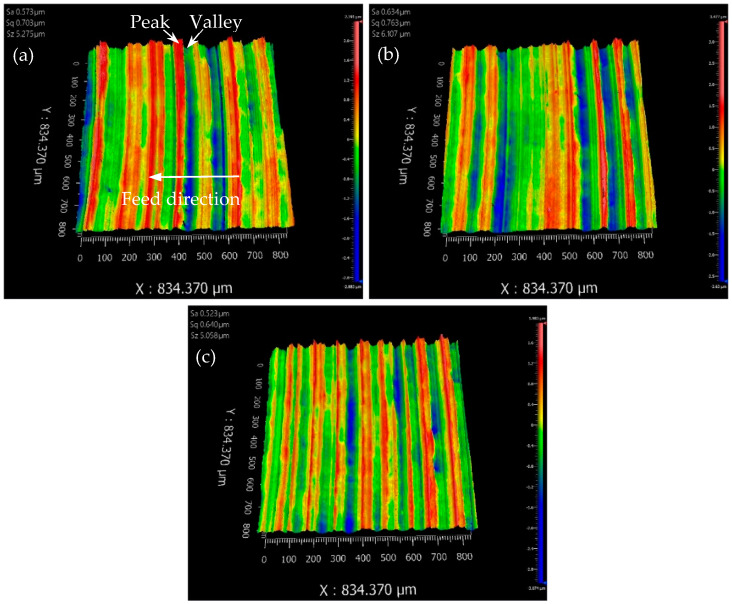
The images of the areal surface roughness of the verified sample 10: (**a**), (**b**), and (**c**) is from the first, the second and the third sampling area, respectively.

According to the direct analysis, A_3_B_1_C_2_ is supposed to be the best combination of the parameters for the smallest values of Sa and Sz. To verify the analysis result, another sample (No. 10) was machined with the combination of A_3_B_1_C_2_, i.e., the cutting speed of 85 m/min, the feed rate of 0.05 mm/rev, and the depth of cut of 0.3 mm. The areal surface topographies from three sampling areas of sample 10 are shown in Figure 4a–c. The visual images give us a direct understanding of the surveyed surface, including tooth marks, feed directions, distance between peak and valley. It is observed that the surface topography in Figure 4c is more consistent than that in Figure 4a,b, which is confirmed by the smallest Sa and Sz compared with the corresponding values from another two images.

The measured values of Sa from the three sampling areas are 0.573, 0.634, and 0.523 µm, respectively. The average of Sa is 0.577 ± 0.056 µm (with SD), which is 1.7% smaller than that (0.594 µm) resulted from A_2_B_1_C_2_. The measured values of Sz from the three sampling areas are 5.275, 6.107, and 5.058 µm, respectively. The average of Sz is 5.480 ± 0.554 µm, which is 28.6% smaller than that (6.923 µm) resulted from A_3_B_1_C_3_. Therefore, the combination of A_3_B_1_C_2_ is verified to be the optimal combination for obtaining minimal Sa and Sz. In the case of the feed rate at the lowest level, higher cutting speed (from level 2 to level 3) results in a slight decrease of Sa and a large decrease of Sz. Lower depth of cut (from level 3 to level 2) results in a relatively large decrease of Sa and a small decrease of Sz. These effects of the two nonsignificant factors on Sa and Sz are consistent with the relationship got from range, ANOVA, and regression analysis.

Moreover, Equation (3) was used to predict the Sa of sample 10 with the parameter values of A_3_B_1_C_2_, to verify the availability of the empirical prediction model. The calculated Sa is 0.596 µm, with the 3% relative error from the measured value (0.577 µm). Therefore, the empirical model of Sa can accurately predict the areal surface roughness Sa of AZ31B after dry face turning with the given machining parameters.

## 4. Conclusions

The effects of different dry turning parameters (cutting speed, feed rate, and depth of cut) on the areal surface roughness Sa and Sz of AZ31B magnesium alloy was investigated in this paper. The following conclusions were drawn from the experimental analysis and linear regression model: 

1. The optimal machining parameters of AZ31B was obtained through the direct analysis of the orthogonal experiment results since a smaller surface roughness means better surface topography. Both Sa and Sz reduce with the decrease in feed rate. The smallest Sa of 0.577 µm and Sz of 5.480 µm were obtained in the verification experiment with the cutting speed at level 3 (85 m/min), feed rate at level 1 (0.05 mm/rev), and depth of cut at level 2 (0.3 mm). The results verify that A_3_B_1_C_2_ is the optimal combination of the machining parameters.

2. Range and ANOVA analysis results show that the feed rate, depth of cut, and cutting speed contribute the greatest, second, and smallest to the value of Sa, respectively. The feed rate, cutting speed, and depth of cut contribute the greatest, second, and smallest to the value of Sz, respectively. Feed rate is the most significant parameter for both Sa and Sz.

3. An empirical model of Sa is established by multiple linear regression analysis. Sa can be accurately predicted with this model since the variation of the three factors can at least account for 97.5% variation of Sa. The availability of this model is confirmed by the verification experiment.

This research provides an approach to explore the optimal combination of dry turning parameters for the smallest areal surface roughness of AZ31B Mg alloy. It also maps the relationship between areal surface roughness and processing parameters. Thus, an experimental framework combined with regression analysis was established to predict the areal surface roughness and get the expected surface topography for biomedical AZ31B subjected to dry face turning.

## Figures and Tables

**Table 1:** Factors and levels of the dry turning parameters.

Level	Factor		
	A Cutting Speed (m/min)	B Feed Rate (mm/rev)	C Depth of Cut (mm)
1	A_1_ = 47	B_1_ = 0.05	C_1_ = 0.2
2	A_2_ = 66	B_2_ = 0.08	C_2_ = 0.3
3	A_3_ = 85	B_3_ = 0.11	C_3_ = 0.4

**Table 2:** The orthogonal experiment design and measurement results.

Sample	Factor			Measured Values of Sa (Sz) ^1^, µm			Averages and SD of Sa (Sz), µm
	A	B	C	Area 1	Area 2	Area 3	
1	1	1	1	0.655 (8.130)	0.698 (8.165)	0.614 (9.791)	0.656 ± 0.042 (8.695 ± 0.949)
2	1	2	2	0.996 (12.691)	0.911 (8.934)	0.884 (11.084)	0.930 ± 0.058 (10.903 ± 1.885)
3	1	3	3	1.521 (16.929)	1.481 (12.951)	1.472 (15.196)	1.491 ± 0.026 (15.025 ± 1.994)
4	2	1	2	0.599 (7.401)	0.521 (6.647)	0.663 (8.973)	0.594 ± 0.071 (7.674 ± 1.187)
5	2	2	3	1.012 (11.338)	0.969 (10.937)	1.109 (11.694)	1.030 ± 0.072 (11.323 ± 0.379)
6	2	3	1	1.590 (12.852)	1.427 (10.934)	1.431 (11.635)	1.483 ± 0.093 (11.807 ± 0.970)
7	3	1	3	0.762 (7.553)	0.571 (7.955)	0.538 (5.260)	0.624 ± 0.121 (6.923 ± 1.454)
8	3	2	1	1.038 (9.638)	0.958 (8.863)	0.970 (10.370)	0.989 ± 0.043 (9.624 ± 0.754)
9	3	3	2	1.467 (12.510)	1.382 (9.562)	1.430 (10.928)	1.426 ± 0.043 (11.000 ± 1.475)

^1^ Data in the brackets are the corresponding value for Sz (the same in the following tables).

**Table 3:** The results of direct analysis and range analysis for Sa and Sz.

Item	Factor		
	A	B	C
$\bar{K1}$	1.026 (11.541)	0.625 (7.764)	1.042 (10.042)
$\;\bar{K2}$	1.036 (10.268)	0.983 (10.617)	0.984 (9.859)
$\bar{K3}$	1.013 (9.182)	1.467 (12.611)	1.048 (11.090)
Range	0.023 (2.359)	0.842 (4.847)	0.065 (1.230)

**Table 4:** ANOVA analysis results of Sa and Sz.

Factor	Square Sum of Dispersion	df ^1^	Mean Square Error	F-Value ^2^	P-Value ^3^
A	0.0008 (8.3657)	2	0.0004 (4.1828)	0.8276 (8844.6160)	0.5472 (0.0001)
B	1.0719 (35.6070)	2	0.5359 (17.8035)	1133.2236 (37645.3894)	0.0009 (0.0000)
C	0.0077 (2.6490)	2	0.0038 (1.3245)	8.0980 (2800.6372)	0.1099 (0.0004)
Experiment Error	0.0009 (1.2064)	2	0.0005 (0.6032)	-	-

^1^ The abbreviation of the degree of freedom. ^2^ The result of the homogeneity test for the variance. ^3^ The probability of samples obtained when the hypothesis is true.

**Table 5:** Coefficients for evaluating the goodness of fit of the regression model of Sa.

R	R^2^	Adjusted R^2^	Standard Error
0.992	0.984	0.975	0.058

**Table 6:** ANOVA analysis results of the multiple regression model.

Model	Sum of Squares	df	Mean Square	F-Value	Significance
Regression analysis	1.064	3	0.355	103.953	0.000
Residual	0.017	5	0.003	-	-
Total	1.081	8	-	-	-

**Table 7:** Coefficients of the multiple linear regression model of Sa.

Item	Non-standardized Coefficient		Standardized Coefficient	t	*P*-Value
	B	Standard Error			
Intercept	−0.084	0.128	-	−0.659	0.539
Cutting speed	−0.00035	0.001	−0.015	−0.266	0.801
Feed rate	14.033	0.795	0.992	17.657	0.000
Depth of cut	0.028	0.238	0.007	0.119	0.910
